# Management of Meige syndrome with bilateral trigeminal and facial nerves combing

**DOI:** 10.3389/fneur.2024.1410531

**Published:** 2024-08-15

**Authors:** Tingting Ying, Haopeng Wang, Yinda Tang, Hua Zhao, Xiaomin Cai, Yiman Shen, Baimiao Wang, Wanchun Zhu, Ping Zhou, Xin Zhang, Jun Zhong, Xinjun Wang, Xudong Fu, Jin Zhu, Weituo Zhang, Shiting Li

**Affiliations:** ^1^Department of Neurosurgery, Xinhua Hospital Affiliated to Shanghai Jiao Tong University School of Medicine, Shanghai, China; ^2^The Cranial Nerve Disease Center, Shanghai Jiao Tong University, Shanghai, China; ^3^Department of Neurosurgery, The Fifth Affiliated Hospital of Zhengzhou University, Zhengzhou, China; ^4^Institute of Neuroscience, Zhengzhou University, Zhengzhou, China; ^5^Hongqiao International Institute of Medicine, Shanghai Tongren Hospital and School of Public Health, Shanghai Jiao Tong University School of Medicine, Shanghai, China

**Keywords:** Meige syndrome, combing, trigeminal nerves, facial nerve, BFMDRS-M

## Abstract

**Objective:**

Meige syndrome (MS) is an adult-onset segmental dystonia for which no satisfactory remedy currently exists. Our team developed a novel surgical approach called bilateral trigeminal/facial nerve combing (BTFC). This study aimed to evaluate the outcomes of patients who underwent BFTC (Clinical Trial Registry Number: ChiCTR2000033481).

**Method:**

We assigned 22 patients with MS to undergo BTFC. The primary outcome was assessed using the movement subscale of the Burke-Fahn-Marsden Dystonia Rating Scale (BFMDRS-M) at 12 months postoperatively. The second outcome was evaluated using the Medical Outcome Study (MOS) 36-item Short Form Health Survey (SF-36), the dysfunction subscale of the Burke-Fahn-Marsden Dystonia Rating Scale (BFMDRS-D), and the sub-item scores of the BFMDRS-M. Safety outcomes included the House-Brackmann (HB) functional grading score and the visual analog scale (VAS) for facial numbness.

**Results:**

At the final follow-up at 12 months, the BFMDRS-M showed a mean improvement of 70.7% from baseline. Mean scores of the BFMDRS-M sub-motor (including the eyes, mouth, and speech/swallowing) improved by 65.6, 81.00, and 60%, respectively. The median score of the total BFMDRS-D score was 0.70 ± 1.17 compared with 1.86 ± 2.21 at baseline. There were no serious operative complications in this population. The quality of life of the patients significantly improved (*P* < 0.05).

**Conclusion:**

BFTC has proven to be effective in relieving the symptoms of Meige syndrome. This novel surgical approach offers a new alternative treatment for patients who have failed to respond to medications, botulinum toxin injections, and deep brain stimulation (DBS).

**Clinical Trial Registration:**

https://www.chictr.org.cn/bin/project/edit?pid=54567, ChiCTR2000033481.

## Introduction

There are currently no curative solutions for Meige syndrome (MS). According to current guidelines, botulinum toxin injection into the facial musculature is the recommended first-line treatment. However, its efficacy diminishes over time, and some patients may develop antibodies that render the therapy ineffective. In addition, botulinum toxin injections can cause weakness in adjacent muscles and may even aggravate pre-existing dysphagia or dysarthria (1). Deep brain stimulation (DBS) is an alternative, but the effective rate is only 45–53% (2). More effective treatments are urgently needed to improve patients' long-term quality of life.

Current theories suggest that the symptoms of MS are closely related to an imbalance of neurotransmitters in the brain and abnormal functional connections between different brain regions, leading to abnormal cranial nerve function in the head and the face. Neurophysiological findings indicate that the blink reflex circuit is hyperexcitable in patients with MS.

We have discovered from thousands of previous microvascular decompressions (MVDs) that a selective comb. of the trigeminal (V) or facial (VII) nerves is feasible (3, 4) as long as intraoperative monitoring ensures that neither the amplitude of the blink reflex R2 nor the amplitude of the facial EMG decreases by more than 30%.

Therefore, we hypothesized that reducing the hyperexcitability of the blink reflex circuit by combing. the cisternal segments of the trigeminal and facial nerves may alleviate the symptoms of patients with MS.

Based on the above hypothesis, our team conducted a pilot study between 2019 and 2020 involving six patients with MS. Three patients underwent unilateral trigeminal/facial nerve combing on the side with prominent symptoms, followed by a second-stage procedure on the other side 3 months later. The other three patients underwent a one-stage BTFC.

After 1 year, the Burke-Fahn-Marsden Dystonia Rating Scale-Movement (BFMDRS-M) scores in the two-stage group decreased significantly compared to the one-stage group, with a mean reduction of 67.4 and 40.0%, respectively.

However, the efficacy and safety of bilateral combing of the facial and trigeminal nerves need to be further investigated. Therefore, we conducted this single-center prospective clinical trial.

## Methods

### Trial design and population

We conducted a single-arm trial involving patients with MS at Xinhua Hospital Affiliated to Shanghai Jiao Tong University School of Medicine from July 2020 to February 2022. Adult patients (40–75 years of age) with primary MS were eligible for inclusion. The key inclusion criteria included: (1) a confirmed diagnosis of MS; (2) the ability to tolerate general anesthesia; and (3) the normal structure of facial and trigeminal nerves as confirmed by an MRI scan. A detailed list of the inclusion and exclusion criteria is available in Supplementary Appendix 1.

The trial protocol was approved by the Institutional Review Board of Xinhua Hospital. The trial was conducted in accordance with the principles of the Declaration of Helsinki and was registered at the Chinese Clinical Trials Registry (ChiCTR2000033481; 2 June 2020). Written informed consent was obtained from all participants prior to enrollment. All listed authors made significant contributions and vouched for the completeness and accuracy of this report and its adherence to the protocol. The trial complied with the CONSORT guideline.

All patients were evaluated by a movement disorders specialist at our center using a video documentation of the preoperative and postoperative neurological assessments. The examiner was not involved in the initial treatment and was blinded to patient information. The BFMDRS-M and disability subscales were used to evaluate the severity of dystonia (5).

### Interventions

#### Standard surgical procedures

**Figure 1:**
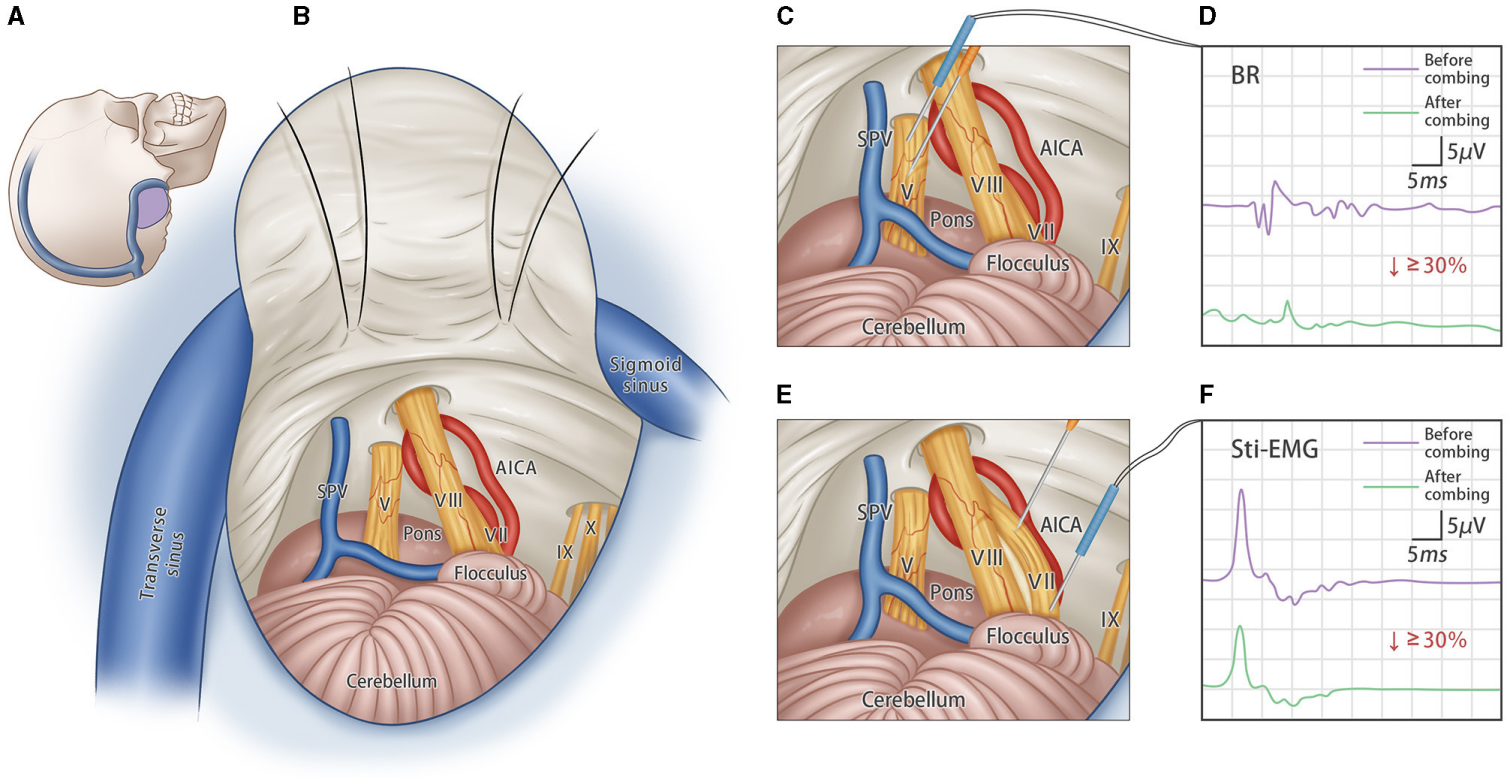
Bilateral trigeminal-facial nerves combing surgery. **(A)** Schematic incision of standard retrosigmoid approach; **(B)** Schematic diagram of intraoperative exposure of important structures, showing the CN-V and CN-VII; **(C)** The sensory fibers of the trigeminal root are combed several times longitudinally by using the custom-made micro-knife. **(D)** A concentration probe was used to stimulate the CN-V nerve directly, and the degree of combing was assessed by changes in the amplitude of the blink reflex (BR) before and after combing. Combing stops when the R2 amplitude of BR decreases by more than 30%. **(E)** The facial nerve is combed several times along the nerve fibers. **(F)** Concentric probe is used to directly stimulate the CN-VII nerve, and the degree of combing is assessed by changes in the amplitude of the stimulate-electromyography (stim-EMG). Combing stops when the amplitude of stim-EMG decreases by more than 30%.

All surgical procedures were performed at Xinhua Hospital Affiliated to Shanghai JiaoTong University School of Medicine. The specific procedure for bilateral trigeminal and facial nerve combing techniques is shown schematically in Figure 1. In brief, short-acting muscle relaxants were used only for tracheal intubation and were not maintained during the operation. Neurophysiological monitoring was employed intraoperatively to measure the amplitudes of the blink reflex (BR) and stimulation-electromyography (stim-EMG) in real time.

The operation was first performed on one side with the patients in a lateral decubitus position using a standard suboccipital retrosigmoid approach. The entire intracranial portion of the trigeminal and facial nerves was exposed. The neurovascular relationship was carefully evaluated to identify any vessels in contact with the nerves. Gelatine sponges were inserted to separate the involved vessels from the nerves. Then, the dorsal third cisternal segment of the trigeminal nerve was combed longitudinally using a custom-made micro-knife (Suzhou, Qimai Inc.). The combing procedure stopped when the amplitude of the blink reflex dropped by 30%. Subsequently, the micro-knife was inserted vertically into the cisternal segment of the facial nerve and combed longitudinally until the amplitude of the stim-EMG dropped by 30%. Afterward, the same procedures were performed contralaterally.

The specific details of intraoperative neurophysiological monitoring are described in Supplementary Appendix 2 and presented schematically in Supplementary Figure 1. All patients received oral drug therapy for 12 months postoperatively, including mecobalamin (0.5 mg tid) and vitamin B12 (20 mg tid), to promote nerve function recovery.

### Outcomes

The primary efficacy endpoint was the movement subscale of the BFMDRS-M at 12 months postoperatively, evaluated by two independent investigators using a video recording of the assessments.

The secondary outcomes were (1) changes from baseline to month 12 in the Medical Outcome Study (MOS) 36-item Short Form Health Survey (SF-36), (2) changes from baseline to month 12 in the disability subscale of the Burke-Fahn-Marsden Dystonia Rating Scale (BFMDRS-D), and (3) changes from baseline to month 12 in sub-item scores of BFMDRS-M, which includes the assessments of the eyes, mouth, speech/swallowing, and neck.

The primary safety endpoint were as follows:

1. The House-Brackmann (HB) functional grading score at 12 months and
2. The visual analog scale (VAS) for facial numbness at 12 months.

Additionally, intraoperative neurophysiological monitoring and its relationship with BFMDRS-M score and safety outcomes were also analyzed.

### Statistical analysis

The trial sample size (*n* = 20) was calculated to ensure 80% power to detect a 10-point (SD = 5) increase in the BFMDRS-M score and to identify any adverse events or other safety outcomes with an expected incidence of at least 8% (6, 7).

An analysis of the primary outcomes was performed using the full analysis set (FAS). For continuous outcomes, the median (95% CI) of the paired difference between baseline and 1 year after surgery was estimated and tested using the Wilcoxon matched-pairs signed rank test. Categorical outcomes were analyzed using McNemar's test. No multiplicity adjustment was applied for secondary endpoints. A sensitivity analysis for the primary outcome was conducted using the per protocol set (PP). Adverse events (AEs) were assessed in the safety set (SS). Multiple imputations were used to address the missing data. All statistical analyses were conducted using R (version 4.2). A *p*-value of < 0.05 (two-sided) was considered statistically significant.

## Results

### Patients

**Figure 2:**
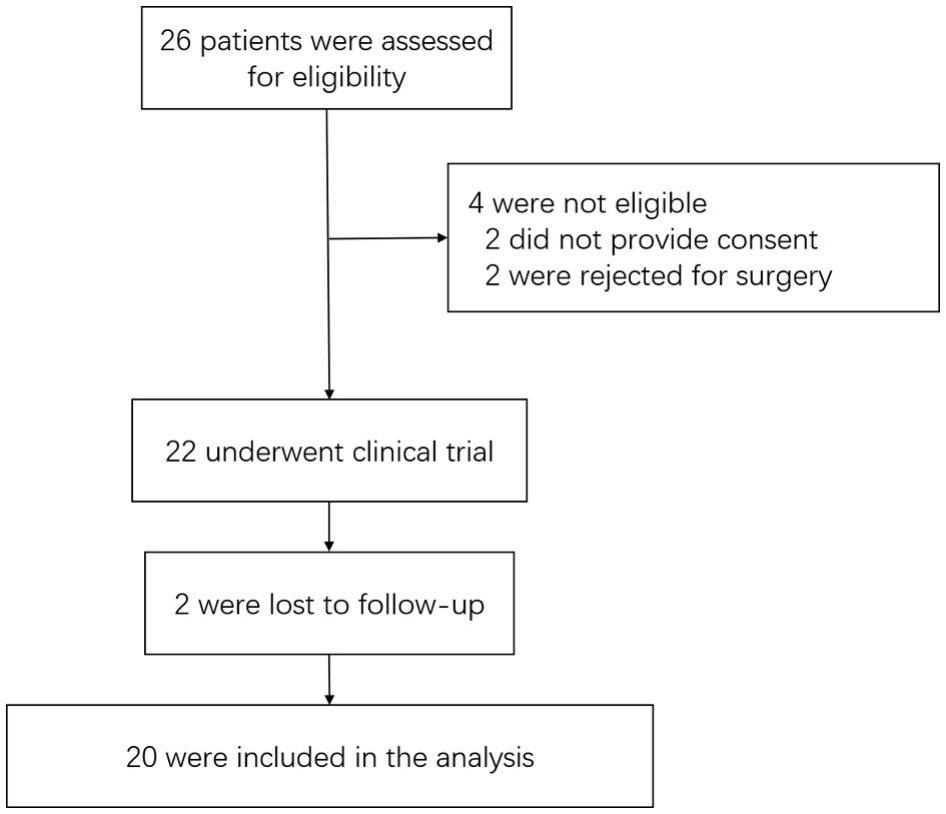
Flow diagram.

From 1 July 2020 and 20 February 2022, 26 patients were screened, of whom 22 were enrolled. Four patients were not enrolled because they either declined to provide consent (two patients) or were unwilling to undergo surgery (two patients). Two of the 22 patients were lost to at postoperative follow-up, resulting in 20 patients being included in the outcome analysis. A flow diagram of the enrollment of patients and follow-up is shown in Figure 2.

The clinical characteristics of the patients are summarized in Table 1. The ages of the patients ranged from 41 to 71 years (median age 58.27 years, IQR 49.00–65.50). The median duration of symptoms was 27.00 months (IQR 12.00–48.00). The baseline data are shown in Table 1.

**Table 1:** Demographic and baseline characteristics of the patients.

**Characteristic**	**Overall (*N* = 22)**
Female sex—no. (%)	16 (72.7)
Median age (IQR)—year	58.27 [49.00, 65.50]
Duration of illness (IQR)—months	27.00 [12.00, 48.00]
**Botulinum toxin (BTX) treatment history—no. (%)**	
Yes	13 (59.1)
No	9 (40.9)
House-Brackmann facial grading system (I grade)—no. (%)	22 (100.0)
Facial numbness score on the VAS (IQR)	1.00 (1.0−1.0)
**Burke–Fahn–Marsden Dystonia Rating Scale (BFMDRS)— (median [IQR])**	
BFMDRS-M (median [IQR])	15.00 [12.25, 16.00]
BFMDRS-D (median [IQR])	0.50 [0.00, 4.00]
**BFMDRS-M subscale**	
BFM-eye (median [IQR])	8.00 [6.50, 8.00]
BFM-mouse (median [IQR])	6.00 [4.00, 6.00]
Speech and swallowing (median [IQR])	0.50 [0.00, 2.75]
Neck (median [IQR])	0.00 [0.00, 0.00]

### Primary endpoint

The BFMDRS scores gradually decreased and reached statistical significance at postoperative periods (1 week, 3, 6, and 12 months after surgery). The mean change in the total BFMDRS-M score at 12 months after surgery was 4.03 ± 2.52 compared to 13.77 ± 4.03 at baseline. This represents a mean improvement of 70.7% in the total BFMDRS-M movement scores 12 months after surgery, showing a significantly greater improvement.

We also analyzed the correlation between the BFMDRS-M score and both age and illness duration. Patients were divided into two groups based on age at onset (< 55 or ≥55 years) and duration of illness (< 2 years or ≥2 years). The mean improvement rates of the BFMDRS-M scores were analyzed 12 months after surgery. The results showed that the postoperative BFMDRS-M score was lower in patients aged < 55 years and those with an illness duration of < 2 years (Supplementary Figure 2).

### Secondary endpoint

**Figure 3:**
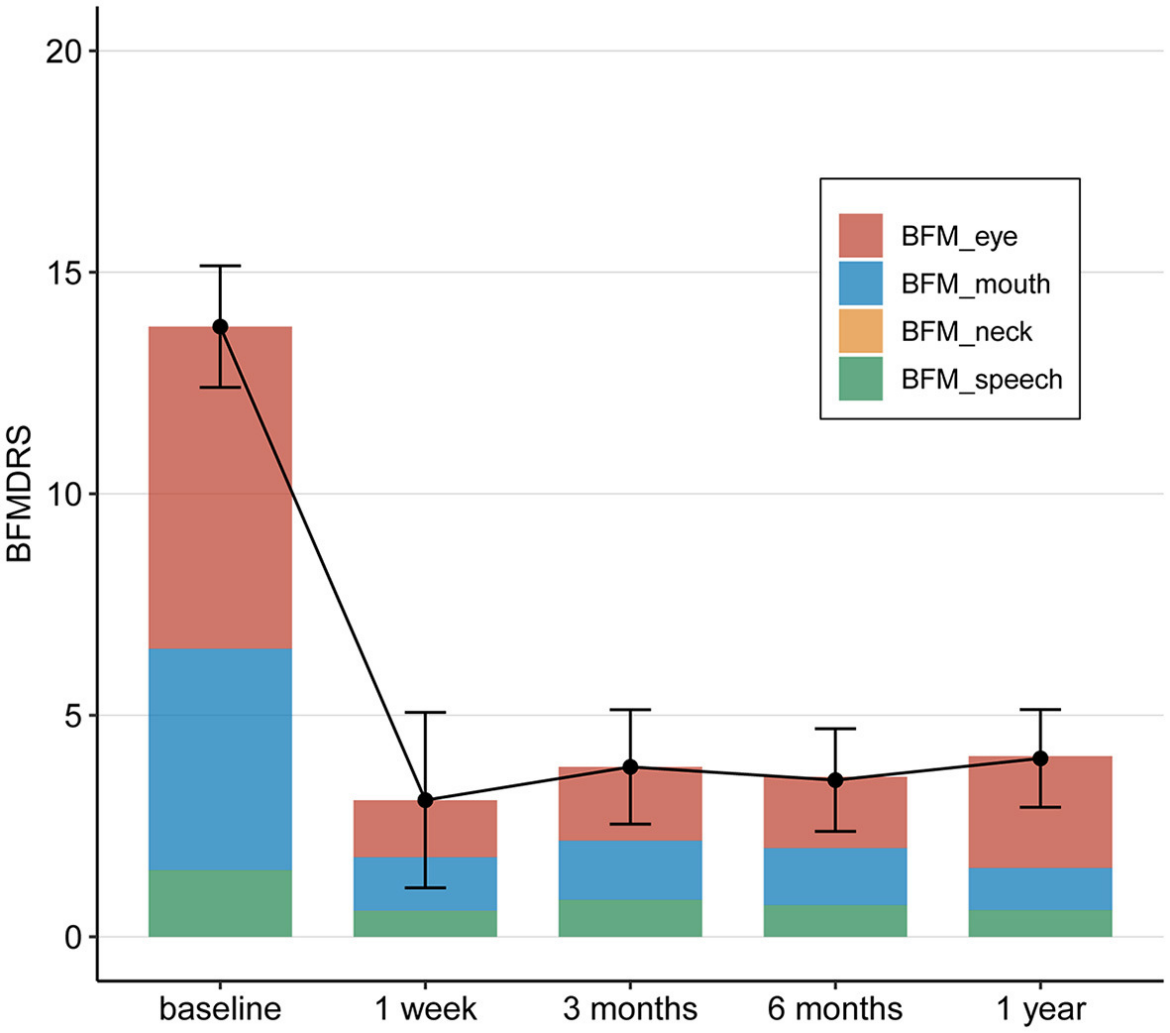
The changes in BFMDRS-M pre-operation and 1 week, 3, 6, and 12 months post-operation.

At month 12 postoperatively, the mean BFMDRS-M sub-item movement scores were as follows: the score for “Eyes” was 2.52 ± 2.09, with an improvement rate of 65.6% (from the baseline score of 7.27 ± 1.32); the score for “Mouth” was 0.95 ± 1.09, with an improvement rate of 81.00% (from the baseline score of 5.00 ± 2.60); the score for “Speech/swallowing” was 0.60 ± 0.88, with an improvement rate of 60% (from the baseline score of 1.50 ± 1.82); and the score for “Neck” was 0.00 (the same as the baseline score, 0.00; Table 2 and Figure 3).

**Table 2:** Primary, secondary, and safety outcomes at 12 months.

**Outcome**	**Before surgery (*N* = 20)**	**After surgery (1 year; *N* = 20)**	***P***
**Primary outcome**			
**Burke–Fahn–Marsden Dystonia Rating Scale (median [IQR])**			
BFMDRS-M	15.0 (11.5–16.0)	4.0 (2.0–6.0)	< 0.001
**Secondary outcomes**			
**BFMDRS-M subscale (median [IQR])**			
BFM-eye	7.27 (1.32)	2.52 (2.09)	< 0.001
BFM-mouse	5.00 (2.60)	0.95 (1.09)	< 0.001
Speech and swallowing	1.50 (1.82)	0.60 (0.88)	0.052
Neck	0.0 (0.0)	0.0 (0.0)	NaN
BFMDRS-D	1.86 (2.21)	0.70 (1.17)	0.042
**MOS item short-form health survey (SD)**			
General health	55.0 (48.8–61.3)	70.0 (60.0–80.0)	< 0.001
Physical functioning	55.0 (48.8–86.3)	85.0 (58.8–96.3)	0.002
Role-physical	25.0 (0.0–37.5)	50.0 (43.8–75.0)	< 0.001
Role-emotional	33.0 (0.0–100.0)	67.0 (33.3–100.0)	0.002
Social functional	50.0 (37.5–62.5)	75.0 (50.0–75.0)	< 0.001
Bodily pain	100.0 (100.0–100.0)	100.0 (100.0–100.0)	0.15
Vitality	50.0 (45.0–75.0)	75.0 (53.8–80.0)	0.001
Mental health	63.3 (52.5–67.5)	66.7 (66.7–71.7)	0.003
**Safety outcomes**			
**House-Brackmann facial grading system—no. (%)**			0.036
I	20 (100)	17 (85)	
II	0 (0)	3 (15)	
Facial numbness score on the VAS (median [IQR])	1.05 (0.21)	1.00 (0.00)	0.347

**Figure 4:**
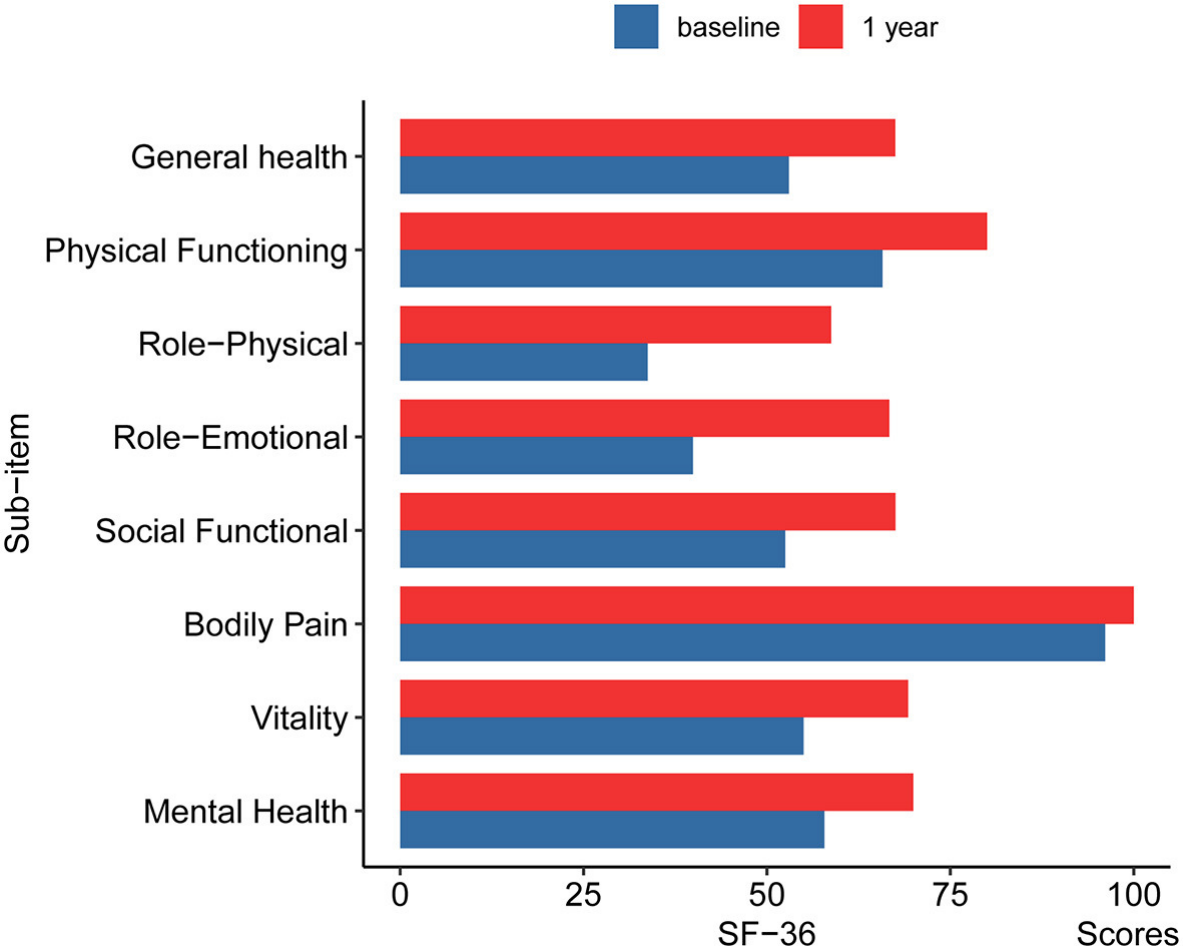
The changes of SF-36 in pre-operation and 12 months post-operation. GH, General health; PF, Physical Functioning; RP, Role-Physical; RE, Role-Emotional; SF, Social Functional; BP, Bodily Pain; VT, Vitality; MH, Mental Health.

The median total BFMDRS-D score was 0.70 ± 1.17 at 12 months after surgery compared to 1.86 ± 2.21 at baseline. According to the SF-36, the patients' quality of life improved significantly at 12 months after surgery (Figure 4). This indicates that the surgery alleviated the symptoms of dystonia. After the surgery, patients were able to resume their daily activities and lifestyle.

### Safety

Adverse events related to the treatment (as determined by the principal investigator) included facial paralysis and facial numbness.

Seven patients reported facial paralysis after surgery, with facial paralysis classified as House-Brackmann (HB) Grade II. After therapy with oral tablets (mecobalamine, vitamin B12, Salvia, nimodipine, and prednisone), two patients recovered to normal within 3 months, and two patients recovered to normal within 6 months. However, three patients continued to experience facial paralysis at HB Garde II 12 months after surgery. Only one patient reported facial numbness after surgery, and this patient recovered to normal within 12 months.

Changes in blink reflex (BR) monitoring of intracranial stimulation and stim-electromyography (stim-EMG) were recorded before and after the combing process. The median decrease in the R2 amplitude of the BR (BR-R2) was 38% (IQR 3–48). The median decrease in the wave amplitude of the stimulated EMG (Stim-EMG) was 41% (IQR 33–47).

The relationship between the degree of decrease in the wave amplitude of the actual intraoperative blink reflex R2 and stim-EMG and the postoperative BFMDRS-M scores was analyzed. The results showed that, as the blink reflex and stim-EMG wave amplitudes decreased during the surgical procedure, the BFMDRS-M scores of the patients also decreased (Supplementary Figure 3A).

We analyzed the relationship between the declining rate of stim-EMG wave amplitude and facial paralysis, as assessed by the House-Brackmann facial grading system. The results indicated that, as the stim-EMG wave amplitude declined, the incidence rate of facial paralysis increased. We also analyzed the relationship between the decline in BR-R2 wave amplitude and the incidence rate of facial numbness. The results showed that, as the BR-R2 wave amplitude declined, the incidence rate of facial numbness increased. It is worth mentioning that, when the BR-R2 and stim-EMG wave amplitudes decreased by more than 45%, patients were more susceptible to symptoms of facial numbness and facial paralysis (Supplementary Figure 3B).

## Discussion

MS is a functionally disabling disease, characterized primarily by blepharospasm and oromandibular dystonia. The onset of MS typically occurs between the ages of 30 and 70 years (with a mean age of 55.7 years) (1, 8). The prevalence of MS is estimated to be ~36–117 per million people (9). Persistently abnormal contraction of the facial muscles can lead to visual impairment and even functional blindness, as well as difficulties in chewing, swallowing, and speaking, severely affecting patients' quality of life.

The exact etiology and pathogenesis of MS remain unclear; however, various hypotheses have been proposed:

1) Dopaminergic and cholinergic hyperactivity (1, 10),
2) dysfunction of the basal ganglia and thalamus leads to an imbalance of dopamine, anticholinergic, and γ-amino acids, resulting in the dysregulation of neural excitation and inhibition (1, 8), and
3) environmental triggers and genetic predisposition cause plastic changes and reduced cortical inhibition (1).

Despite the unclear pathogenesis of MS, patients with MS exhibit similar clinical manifestations, including varying degrees of eyelid and facial muscle twitching, blinking difficulties, and frequent facial involuntary movements (2, 8). A study by Schwingenschuh et al. found that the R2 recovery cycle was significantly disinhibited in patients with MS (11). Additionally, a clinical, electrophysiological study by Akalin et al. found that patients with MS had the highest mean R2 amplitude and duration values compared to the healthy control group (12). All of these previous studies have shown that the blink reflex circuit is abnormally excited in patients with MS (5).

Based on the observation of abnormal hyper-excitability in the BR circuit, our team developed a new surgical approach called BTFC. This novel treatment aims to improve the abnormal spasticity of facial muscles in patients with MS to reduce the abnormal hyper-excitability of the BR circuit by combing the facial and trigeminal nerves. In a follow-up study involving 20 patients with MS, the total BFMDRS-M score decreased by 70.7% at 12 months after surgery, indicating that BTFC can effectively reduce the symptoms of patients with MS. The degree of improvement in motor symptoms was comparable to that achieved with DBS (6).

However, DBS surgery has several disadvantages, including a long treatment period and the necessity of implanting foreign objects, such as batteries and electrodes, which increases the risk of infection and rejection (7).

As the extent of nerve combing increases during surgery, the degree of symptomatic relief of MS improves, but the risk of facial and trigeminal nerve injury also increases simultaneously. Therefore, balancing the surgical outcome with the risk of facial and trigeminal nerve injuries is important. In our study, we found that reducing the amplitude of intraoperative BR-R2 and facial nerve stim-EMG by 30–45% provided an effective and safer range. Amplitude decreases of < 30% were insufficient to achieve satisfactory surgical results, while amplitude decreases of more than 45% increased the risk of postoperative facial paralysis and facial numbness.

Nevertheless, the optimal degree of nerve combing through electrophysiology remains challenging. One patient experienced mild facial numbness after surgery but returned to normal within 12 months. Additionally, 35% (7/20) of the patients had Grade II mild facial palsy symptoms after surgery. After treatment with methylcobalamin and vitamin supplements, facial nerve function returned to normal in most patients (57.1%) within 12 months after surgery, which may also be attributed to the natural recovery process of the facial nerve. Although a few patients continued to experience mild facial palsy symptoms, these symptoms did not have a significant negative impact on their daily lives. This finding also highlights the importance of minimizing the degree of nerve combing to reduce complications.

We also found that BTFC showed varying rates of symptomatic improvement among different patients. Specifically, the improvement of MS was more pronounced in patients with a disease duration of < 2 years compared to those with more than 2 years. A study on pallidal-DBS for MS indicated that the course of the disease was negatively associated with postoperative outcomes. Patients with a shorter disease duration experienced a faster onset and longer duration of postoperative effects, and shorter disease duration predicted better clinical outcomes (13). This finding is consistent with our findings.

Younger patients with MS appeared to have better outcomes after surgery. Similarly, a study on DBS in the treatment of MS showed that younger patients with shorter durations of symptoms had better clinical outcomes (13). However, the limited number of cases in this study suggests that more research is needed to completely understand the factors influencing prognosis.

In addition, the nerve combing technique has been widely employed in treatments for other disorders. Our previous studies have demonstrated that combing. the trigeminal or facial nerves effectively and safely treats intractable trigeminal neuralgia and facial spasms (3, 4). Posterior spinal nerve rhizotomy (SPR) can be used to treat limb spasticity by adjusting patient's muscle tone so that the spastic muscles become normal. However, the severed nerve in SPR cannot regenerate, and excessive severance can cause irreparable damage (14, 15). In contrast, nerve combing reduces nerve excitability without completely dissecting the nerve. It is safer under electrophysiological monitoring, making it a useful procedure for patients with limb spasticity and cerebral palsy.

## Conclusion

Our study showed that BTFC is an effective and safe treatment for MS. In particular, it provides a new alternative for patients who have failed to respond to medications, botulinum toxin injections, and DBS. Consequently, BTFC is recommended as a second-line treatment option for this disease.

## Data availability statement

The original contributions presented in the study are included in the article/Supplementary material, further inquiries can be directed to the corresponding authors.

## Ethics statement

The studies involving human participants were reviewed and approved by the Ethics Committee of Xinhua Hospital (No. XHEC-C-2020-029). The participants provided their written informed consent to participate in this study.

## Author contributions

TY: Conceptualization, Data curation, Investigation, Methodology, Software, Validation, Writing – original draft, Writing – review & editing. HW: Conceptualization, Data curation, Investigation, Methodology, Software, Writing – original draft, Writing – review & editing. YT: Conceptualization, Investigation, Validation, Writing – review & editing. HZ: Conceptualization, Investigation, Validation, Writing – review & editing. XC: Investigation, Validation, Writing – review & editing. YS: Investigation, Writing – review & editing. BW: Investigation, Validation, Writing – review & editing. WZhu: Investigation, Validation, Writing – review & editing. PZ: Investigation, Validation, Writing – review & editing. XZ: Investigation, Validation, Writing – review & editing. JZho: Investigation, Validation, Writing – review & editing. XW: Investigation, Validation, Writing – review & editing. XF: Investigation, Validation, Writing – review & editing. JZhu: Data curation, Funding acquisition, Investigation, Methodology, Software, Validation, Writing – review & editing. WZha: Data curation, Funding acquisition, Investigation, Methodology, Software, Validation, Writing – review & editing. SL: Data curation, Funding acquisition, Investigation, Methodology, Project administration, Software, Supervision, Validation, Writing – review & editing.
