# Supplementary material for: Management of Meige syndrome with bilateral trigeminal and facial nerves combing
Source: Front Neurol. 2024 Aug 15;15:1410531. doi: 10.3389/fneur.2024.1410531 (PMC11358068; doi:10.3389/fneur.2024.1410531)
Supplement: Supplementary file 1 [file Data_Sheet_1.docx]

**Supplementary Appendix 1: Inclusion and exclusion criteria (V1.0)**

Criteria for inclusion

(1). Definite diagnosis of idiopathic cranial cervical dystonia (blepharospasm, lower facial and oromandibular dystonia, with or without the involvement of cervical muscles).

(2).40-75 years old.

(3). Failed botulinum toxin therapy.

(4). Normal structure and function with facial nerve and trigeminal nerve in MRI.

(5). Patients can tolerate general anesthesia operation.

(6). Patients can describe their subjective feelings.

(7). Willing to receive clinical trial to achieve further functional improvement.

Criteria for exclusion

(1). Had cognitive impairments, dementia or serious psychiatric disorders.

(2). With other dystonia disorders.

(3). Had a history of alcohol or drug abuse.

(4). Brain MR/CT imaging demonstrated structural causes(tumor/trauma) for their dystonia.

(5). With botulinum toxin treatment within 3 months before enrollment.

**Supplementary Appendix 1: Inclusion and exclusion criteria (V1.2)**

Criteria for inclusion

(1). Definite diagnosis of idiopathic cranialcervical dystonia (blepharospasm, lower facial and oromandibular dystonia, with or without the involvement of cervical muscles).

(2).40-75 years old.

(3). Normal structure and function with facial nerve and trigeminal nerve in MRI.

(4). Patients can tolerate general anesthesia operation.

(5). Patients can describe their subjective feelings.

(6). Willing to receive clinical trial to achieve further functional improvement.

Criteria for exclusion

(1). Had cognitive impairments, dementia or serious psychiatric disorders.

(2). With other dystonia disorders.

(3). Had a history of alcohol or drug abuse.

(4). Brain MR/CT imaging demonstrated structural causes(tumor/trauma) for their dystonia.
