# Supplementary material for: Management of Meige syndrome with bilateral trigeminal and facial nerves combing
Source: Front Neurol. 2024 Aug 15;15:1410531. doi: 10.3389/fneur.2024.1410531 (PMC11358068; doi:10.3389/fneur.2024.1410531)
Supplement: Supplementary file 2 [file Data_Sheet_2.docx]

**Supplementary Appendix 2: Intraoperative neurophysiological monitoring**

Recording electrodes for intraoperative electrophysiological monitoring were placed on the bilateral frontalis muscle, orbicularis oculi, orbicularis and mentalis muscles. After the cisternal segment of trigeminal was fully exposed, concentric probe was used to stimulate the distal trigeminal nerve to acquire baseline of blink reflex. The stimulus frequency was 1.0 Hz, the wave width was 0.1ms, and the stimulus intensity was the minimum stimulus intensity that could induce R2 wave of blink reflex. After combing, trigeminal nerve was stimulated again and the decreased amplitude of blink reflex was recorded. It should be noted that the stimulation site was the same and located at the distal end of the combing site. Next, the same concentric probe was used to directly stimulate the proximal facial nerve to obtain the baseline, the stimulus frequency was 1.0Hz, the wave width was 0.1ms, and the stimulus intensity was the minimum stimulus intensity that could stable induce electromyography (with the amplitude greater than 50uv). Facial nerve was stimulated again after combing at the same stimulated site. The amplitude of the two records were compared (Figure S1).
