# Supplementary material for: Management of Meige syndrome with bilateral trigeminal and facial nerves combing
Source: Front Neurol. 2024 Aug 15;15:1410531. doi: 10.3389/fneur.2024.1410531 (PMC11358068; doi:10.3389/fneur.2024.1410531)
Supplement: Supplementary file 3 [file Data_Sheet_3.docx]

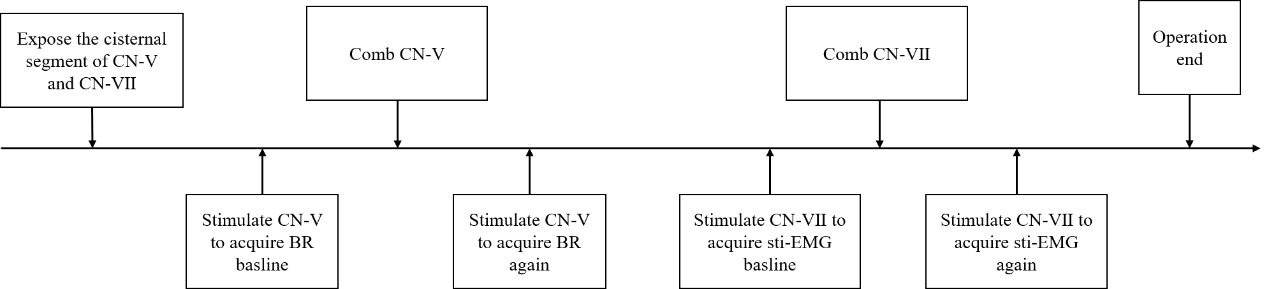
**Figure S1: Flow chart of intraoperative facial and trigeminal nerve combing and electrophysiologic monitoring**

**
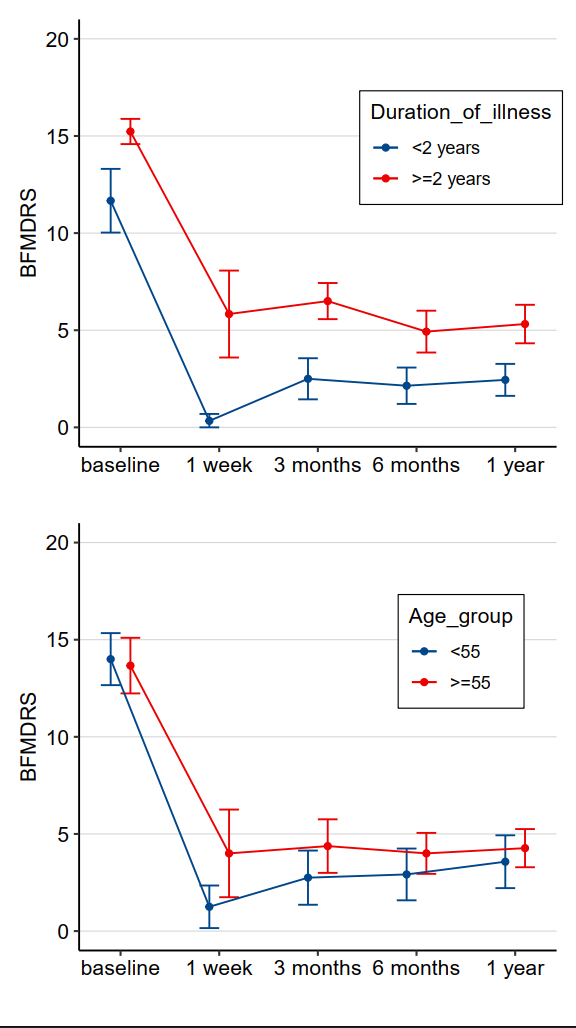
**

**Figure S2: The relationship of outcomes between age onset and illness durations.**

(A) The relationship of BFMDRS-M and illness duration (illness duration was divided into <55 or ≥55 years). (B) The relationship of BFMDRS-M and age onset (age onset was divided into <2 years or ≥2 years).


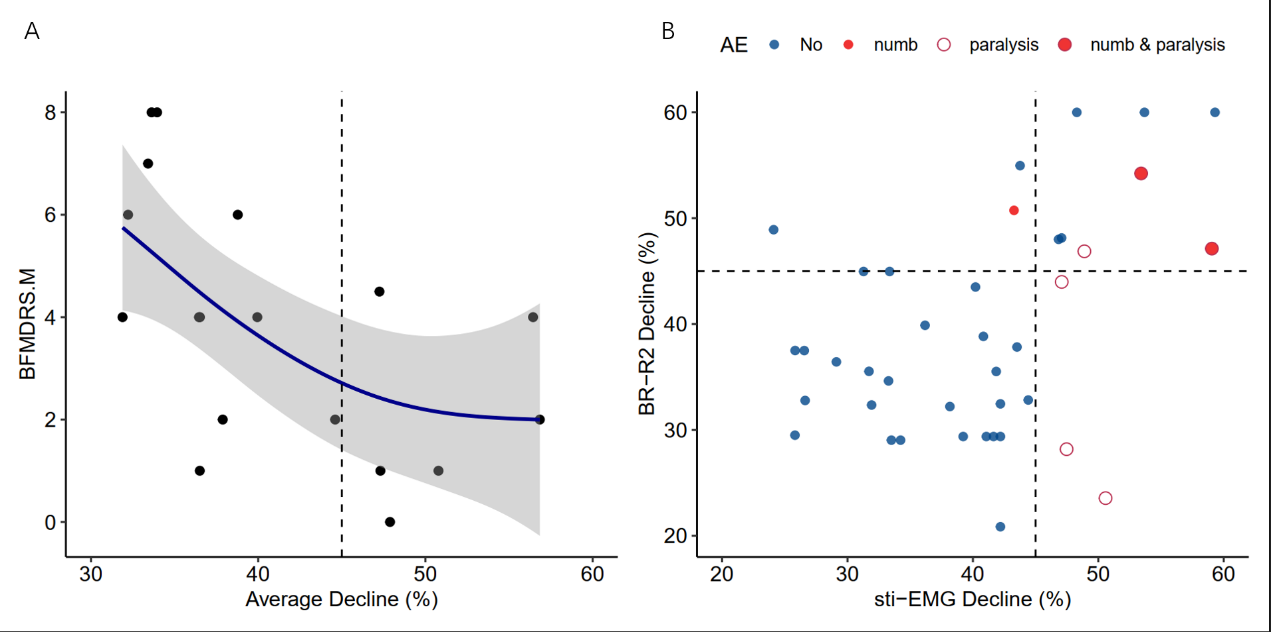
**Figure S3: The relationship between intraoperative neuromonitoring and postoperative BFMDRS-M score.** (A) The greater the reduction in wave amplitude of the blink reflex and sti-EMG intraoperatively, the higher the postoperative BFMDRS-M score. (B) when the amplitude of blink reflex R2(BR-R2) and sti-EMG decreased more than 45%, patients are susceptible to facial numbness and facial paralysis symptoms (Figure S4B).
